# Effect of the Processing Parameters on the Porosity and Mechanical Behavior of Titanium Samples with Bimodal Microstructure Produced via Hot Pressing

**DOI:** 10.3390/ma15010136

**Published:** 2021-12-25

**Authors:** Ricardo Chávez-Vásconez, Sheila Lascano, Sergio Sauceda, Mauricio Reyes-Valenzuela, Christopher Salvo, Ramalinga Viswanathan Mangalaraja, Francisco José Gotor, Cristina Arévalo, Yadir Torres

**Affiliations:** 1 Departamento de Ingeniería Mecánica, Universidad Técnica Federico Santa María, Avenida Vicuña Mackenna 3939, Santiago 8940572, Chile; ricardo.chavezv@usm.cl (R.C.-V.); sergio.sauceda@usm.cl (S.S.); mauricio.reyes@usm.cl (M.R.-V.); 2 Departamento de Ingeniería Mecánica, Facultad de Ingeniería, Universidad del Bío-Bío, Avda. Collao 1202, Casilla 5-C, Concepción 4081112, Chile; csalvo@ubiobio.cl; 3 Departamento de Ingeniería de Materiales, Universidad de Concepción, Edmundo Larenas 270, Concepción 4070409, Chile; mangal@udec.cl; 4 Instituto de Ciencia de Materiales de Sevilla (CSIC-US), Américo Vespucio 49, 41092 Sevilla, Spain; francisco.gotor@icmse.csic.es; 5 Departamento de Ingeniería y Ciencia de los Materiales y del Transporte, Escuela Poliécnica Superior, Calle Virgen de África 7, 41011 Seville, Spain; carevalo@us.es (C.A.); ytorres@us.es (Y.T.)

**Keywords:** porous titanium, bimodal microstructure, hot-pressing, powder metallurgy, mechanical milling, mechanical behavior

## Abstract

Commercially pure (c.p.) titanium grade IV with a bimodal microstructure is a promising material for biomedical implants. The influence of the processing parameters on the physical, microstructural, and mechanical properties was investigated. The bimodal microstructure was achieved from the blends of powder particles with different sizes, while the porous structure was obtained using the space-holder technique (50 vol.% of ammonium bicarbonate). Mechanically milled powders (10 and 20 h) were mixed in 50 wt.% or 75 wt.% with c.p. titanium. Four different mixtures of powders were precompacted via uniaxial cold pressing at 400 MPa. Then, the specimens were sintered at 750 °C via hot pressing in an argon gas atmosphere. The presence of a bimodal microstructure, comprised of small-grain regions separated by coarse-grain ones, was confirmed by optical and scanning electron microscopies. The samples with a bimodal microstructure exhibited an increase in the porosity compared with the commercially available pure Ti. In addition, the hardness was increased while the Young’s modulus was decreased in the specimens with 75 wt.% of the milled powders (20 h).

## 1. Introduction

The research field related to biomedical materials has been grown in recent decades as a result of the demand for implants for bone replacement [1]. Titanium and its alloys are considered the most suitable option for biomedical applications due to their low density, high biocompatibility, specific mechanical strength, and corrosion resistance, as well as their in vitro and in vivo acceptable behavior [2,3]. However, there is mismatch between the stiffness of bone and metallic biomaterials. This incompatibility generates the stress-shielding phenomenon which promotes bone resorption at the implant–bone interface and can even induce implant failure [4,5]. 

It is well known that porous structures exhibit lower elastic modulus than their fully-dense counterparts [3,6]. It has also been identified that titanium components that possess optimal macro/micro porosities allow for the tuning of the elastic modulus in a considerably wide range, which also favors bone cell ingrowth and vascularization [3]. 

Among the manufacturing processes employed to obtain porous metallic materials are tied freeze casting [7,8], selective laser melting (SLM) [9,10], field assisted sintering (FAST) [11,12], and powder metallurgy [13,14]. Powder metallurgy, in combination with the space-holder technique, represents a cost-effective and flexible way to obtain components with a high-degree of porosity (35–80%) and a homogeneous distribution of pores throughout the volume [15,16,17]. Particles commonly used as space-holders include NH_4_HCO_3_ [5,18,19,20,21,22], NaCl [17,18,21,23,24], starch [25,26], Mg [27,28,29], PMMA [30,31], saccharose crystals [26,32], PVA [33], and carbamide [15,34,35], which can be eliminated at a relatively low temperature, or can be easily removed by a dissolution process, generally in water [3]. NH_4_HCO_3_ is one of the preferred spacer particles due to its moderate decomposition temperature, which makes it easily and completely removable, ensuring a low uptake of impurities such as oxygen, nitrogen, and carbon [17].

However, although this increase in porosity leads to a reduction in Young’s modulus, it also reduces mechanical strength. Previous work [36] has shown that, to obtain Young’s modulus close to that of human bone, porosity percentages greater than 45% are required. This leads to a drastic reduction in mechanical resistance, below what is required for bone replacement [17,36,37].

Concerning the mechanical performance, titanium components that exhibit a fine grain structure have gained attention due to the grain boundary strengthening effect [38,39], which results in an important increase in strength and hardness when compared to their coarse-grained counterparts. However, regardless of the processing methods, fine-grained metallic materials usually suffer from poor plastic deformation at room temperature [40]. An approach based on tailoring the microstructure by the development of bimodal/multimodal grain size distributions has been implemented, aiming to optimize the balance between ductility and strength [41,42]. Thus, fine grains provide a strength increase, while coarse grains allow ductility to be retained [43]. This method presents the potential to produce materials with a porous structure and suitable combination of elastic modulus, strength, and ductility, which allows an adequate balance between biological and mechanical behavior for biomedical applications.

In recent investigations, titanium samples with a bimodal microstructure synthesized by spark plasma sintering (SPS) have shown yielding stress and ultimate tensile strength values that exceeded twice the value of conventional α-titanium coarse-grained components [44] and hardness that exceeded the nominal value of commercially pure (c.p.) titanium by 3–4 times [45]. Hot consolidation techniques, such as hot pressing sintering (HP) [46], hot isostatic pressing (HIP) [47], and SPS [45], allow the fabrication of components with bimodal structure due to their characteristics of rapid heat/cooling rates and low sintering temperatures, which limits the excessive grain growth preserving the fine-grained microstructure when compared to the conventional sintering processes [48,49]. Besides, it has also been shown that HP increases the chemical homogeneity of the phases present in titanium alloys, and effectively controls grain growth in their microstructure [50,51]. However, the formation of porous structures, which is essential for Ti implants, by pressure-assisted techniques is rather complicated. To circumvent this problem, the use of the space-holder methodology is proposed in this work. 

Therefore, the aim of this research is to obtain porous samples of titanium with bimodal microstructure via powder metallurgy from blends of powder particles with different sizes, using NH_4_HCO_3_ as a temporary spacer particle, and consolidated by hot pressing. The effect of the processing parameters on the bimodal microstructure, porosity, and microhardness of titanium samples is studied. 

## 2. Materials and Methods

### 2.1. Starting Materials Preparation and Characterization

C.p. titanium grade IV was used as raw powder to produce the bimodal microstructure. According to the supplier’s information (Alfa Aesar, Tewksbury, MA, USA), the mean particle size was less than 45 µm. In order to form the bimodal microstructure, the size and morphology of titanium powders were modified by mechanical milling in a planetary mill RETSCH^®^ PM 400 (Retsch, Haan, Germany). Thus, 20 g of titanium powder, ZrO_2_ (YSZ) ceramic balls with 5 and 10 mm of diameter (ball to powder weight ratio 10:1), and a 2 wt.% of stearic acid as processing control agent (PCA) were placed in a 250 cm^3^ ZrO_2_ vial. One of the studied parameters was the influence of milling time over the obtained bimodal microstructure; thus, the milling was done using two effective milling times: 10 and 20 h. The milling procedures were carried out at 250 rpm, in cycles of milling and resting of 30 min, in argon atmosphere (ultrapure with <3 ppm O_2_) to prevent excessive heating and the oxidation of the powder, respectively. From here, the resulting powders are named Ti_10_ and Ti_20_.

Regarding the amount of fine powder, two different blends of powders were established: 50 wt.% and 75 wt.% of milled powder, where the remaining percentage corresponds to the as-received Ti powder. The processing parameters were chosen in order to detect improvements related to the capability of retaining porosities, although the high densification technique and the mechanical properties achieved this due to the hardening effect during milling stage. Table 1 summarizes the powder parameters used in order to produce each blend. Hence, the four blends are named Ti_10–50_, Ti_10–75_, Ti_20–50_, and Ti_20–75_. 

Before the consolidation stage, morphological analysis of starting powders and NH_4_HCO_3_ particles was carried out by Scanning Electron Microscopy (SEM) with a JSM-6380LV SEM JEOL (JEOL Ltd., Tokyo, Japan) microscope equipped with an Energy Dispersive X-Ray Spectroscopy (EDS) device, according the ASTM F1877 [52] standard. A particle size analysis of the milled and as-received Ti powders were performed using laser diffraction analysis in an Analysette 22 (Frisch GmbH, Idar-Oberstein, Germany) equipment, whereas the granulometric test of NH_4_HCO_3_ study was accomplished by sieving on an SS3 Gilson^®^ (Gilson Incorporated, Global Headquarters, Middleton, WI, USA) device, according to ASTM E2651 standard [53]. Furthermore, X-ray diffraction (XRD) analysis was performed on the as-received and milled (Ti_10_ and Ti_20_) powders. The XRD patterns were obtained with STOE STADI MP (STOE & Cie GmbH, Darmstadt, Germany) using CuKα1 radiation (λ = 0.15406 nm) and a step size of 0.12°; the scan was recorded in the 2θ range comprised from 20° to 120. Once the starting powders were characterized, the specimens were consolidated in order to study the effect of the processing parameters on the final properties of the sintered samples.

### 2.2. Green Specimens Preparation and Hot Consolidation

Before consolidation of specimens, powder blends (Table 1) and NH_4_HCO_3_ (50 vol.%) particles were mixed for 40 min in a TURBULA^®^ T2F (WAB, Muttenz, Switzerland) to reach good homogenization. The amount of prepared mixture was stablished regarding the final dimensions of the specimens, having a cylindrical geometry with a diameter of 12.7 mm and height of 20 mm, according to ISO 13314 [54] and ASTM E9 [55] standards. Next, uniaxial cold compaction of mixtures was performed in a universal testing machine Zwick/Roell Z100 (Zwick/Roell, Ulm, Germany) in two stages. The first one was at a compaction pressure of 20 MPa (3 mm/min) and the second one at 500 MPa (5 mm/min), using 2 min dwell time and 15 min unloading time.

Subsequently, specimens were sintered in a hot press HP20-4560-20 (Thermal Technology LLC, Santa Rosa, CA, USA) in two stages: first, at 100 °C for 1 min in vacuum (10^−2^ mbar), and then at 750 °C and 15 MPa pressure for 15 min in an argon atmosphere (heating rate in both stages was 10 K/min). A graphite die was used, previously coated by a boron nitride-sprayed film to avoid direct contact between the die and the green specimen. The first stage was performed to remove impurities and humidity, and to start the elimination of the space-holder particles, and the second one to continue removing remnants of the spacers and to sinter the green specimens. After sintering, the titanium samples were ground to remove the boron nitride residues. Then, the samples were characterized in order to study the microstructural aspects, porosity, and mechanical properties achieved after the sintering stage.

### 2.3. Microstructural and Mechanical Characterization of Sintered Samples

The specimens were prepared for microstructural analysis and microhardness measurements by conventional steps of metallographic preparation, according to ASTM E3 [28], with a final step consisting of a mechanical-chemical polishing using colloidal silica and hydrogen peroxide.

Size, morphology and porosity distribution, pores roughness, and bimodal microstructure were analyzed by image analysis (IA) of the micrographs obtained by optical microscopy (OM) in a Nikon Eclipse MA100N (Nikon Corporation, Tokyo, Japan) microscope, and by SEM in a Quanta FEG-250 SEM (Thermo Fisher Scientific, Waltham, MA, USA). IA was performed using Image Pro Plus software. The evaluated and studied porosity parameters were: (i) total porosity (*P*(IA)), (ii) equivalent diameter of pores (*D*_eq_), and (iii) pore shape factor (*F_f_* = 4πA/(PE)^2^), where A is the pore area and PE is its perimeter. An *F_f_* value close to 1 suggests a rounded pore, while a value close to 0 suggest a needle-shaped pore.

An analysis of variance (ANOVA) was carried out by a Tukey’s test using Statgraphics^®^ software, considering a significance level of *p* < 5%.

The microhardness measurements were performed in an HMV-G (Shimadzu, Kyoto, Japan) tester, applying a 98.07 mN load for 10 s.

Young’s modulus, $E_{p}$, was estimated by the Nielsen’s equation [56], expressed as follows:

(1) $$E_{p}=E_{Ti}\times \left[\frac{{\left(1-\frac{P\left(\mathrm{IA}\right)}{100}\right)}^{2}}{1+\left(\frac{1}{F_{f}}-1\right)\frac{P\left(\mathrm{IA}\right)}{100}}\right]$$

where, $E_{Ti}$ is the Young’s modulus for c.p. Ti grade IV bulk (~110 GPa [57]), P(IA) is the percentage of total porosity of the sample, and $F_{f}$ is the shape factor calculated from the results of the image analysis.

In addition, the yield strength values were assessed from the correlation proposed by Jha et al. [58], expressed as follows:

(2) $$\sigma_{y,f}=0.74\times \sigma_{y,b}{\left(\frac{\rho_{f}}{\rho_{b}}\right)}^{2.206}$$

where, $\sigma_{y}$ is the yield strength, ρ is the material’s density, and the subscripts f and b are for the porous and bulky material, respectively. The density values were estimated from the measured values of mass and volume of sintered specimens, and the yield strength and density of Ti used were the ones provided by the raw material supplier. This is a preliminary approach to estimate the yield strength due to the employed model and did not consider the effect of bimodal microstructure.

## 3. Results and Discussion

### 3.1. Characterization of Starting Materials

**Figure 1:**
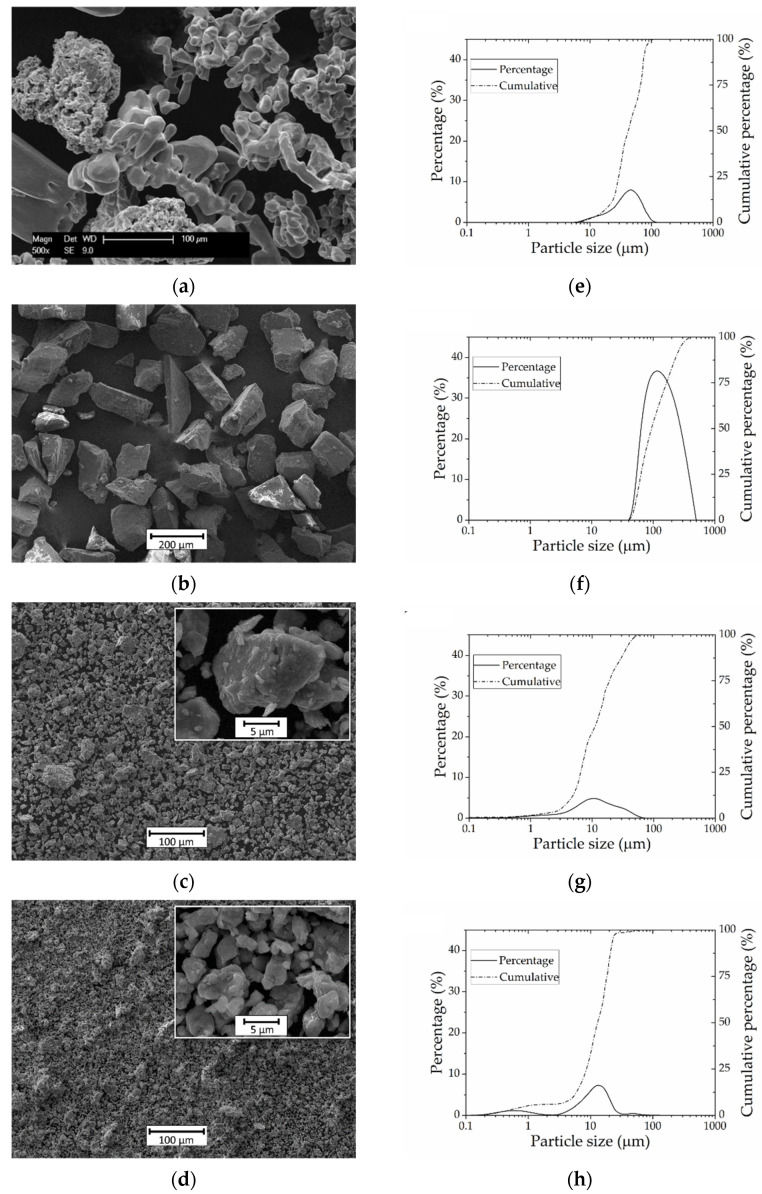
SEM images of: (**a**) as-received titanium powders; (**b**) NH_4_HCO_3_ particles; (**c**) Ti_10_ milled powder; (**d**) Ti_20_ milled powder. Particle size distribution of: (**e**) as-received titanium powder; (**f**) NH_4_HCO_3_ particles; (**g**) Ti_10_ milled powder; (**h**) Ti_20_ milled powder.

The results of the morphological analysis of the starting powders by SEM images and the particle size distribution, obtained by laser diffraction analysis, are depicted in Figure 1. The SEM images of the as-received titanium powders are shown in the Figure 1a, where an irregular shape is appreciated, typically from its processing via hydrogenation/dehydrogenation. The mean particle size is 50 µm in a range of 10–100 µm (Figure 1e). NH_4_HCO_3_ particles, Figure 1b, exhibit a polygonal and cubic morphology with a high dispersion of particle size. Particle size distribution of spacer present a normal distribution with a particle size between 50 µm and 400 µm (Figure 1); this distribution was the result of the sieving stage performed during preparation. Some investigations have shown the relevance of spacer particle size and morphology to generate a structure where the total porosity is controlled in an appropriate manner [17,36,59].

Morphologies and the particle size of milled titanium powders, after 10 h (Ti_10_) and 20 h (Ti_20_), are presented in Figure 1c,g and Figure 1d,h, respectively. In the case of milled Ti, a reduction in particle size and changes in the particle size distribution are obtained as a consequence of the mechanical milling. A mean particle size of 10 µm in the range between 3 and 70 µm was confirmed for Ti_10_ and a bimodal distribution was evidenced for the Ti_20_ with two mean sizes: 15 µm in the 3–30 µm range, and 0.6 µm in a range of 0.2–2 µm. Despite the increase in milling time, the mean particle size of the main distribution of Ti_20_ powders was larger than that of the Ti_10_ powders. This is due to the fact that, in the initial stage of milling, the dominant phenomenon is fracture, whereas, after increasing milling time, the active phenomena are fracture and cold welding [60], which results in an agglomeration of particles and the formation of two distributions for the Ti_20_ powders. This phenomenon has been recognized as the final stage of the particle–particle interactions in dry-milling processes [61]. 

**Figure 2:**
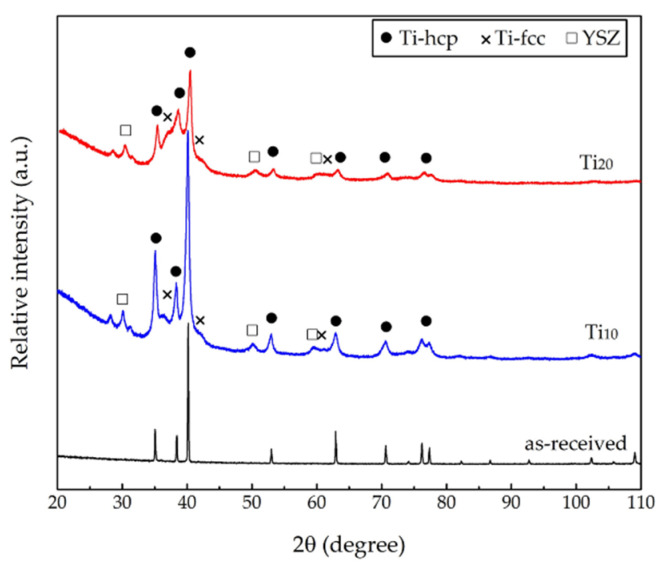
XRD patterns of titanium powders: as-received (black), Ti_10_ (blue), and Ti_20_ (red).

The XRD patterns of the as-received and milled Ti powders are shown in Figure 2, providing structural information. In the case of the as-received powder, only diffraction peaks that correspond to the Ti-hcp phase were detected. On the other hand, both milled powders (Ti_10_ and Ti_20_) exhibited the Ti-hcp peaks, accompanied by several peaks and a “hump” visualized as 2θ = 35°– 42°. The Ti-hcp peaks of both milled powders are broader in shape than the Ti-hcp peaks of the as-received powders, which suggest that, after milling, the crystallite size of this phase may decrease. This effect is frequently observed in high-energy milled powders due to the continuously welding and fracture processes occurring between them during the process [62]. Studying the pattern of both milled powders in detail, the diffraction peaks of Ti-γ were identified (in 2θ = 36.2°, 42.1°, and 61.2°), which is a metastable phase (FCC) with the Fm3m space group, which should not be confused with the Ti-β phase, which is also a cubic structure (BCC) but has an Im3m space group and should have diffraction peaks in 2θ = 38.5°, 55°, and 69°, peaks that are missing in the pattern. It has been reported that the formation of this metastable phase by ball-milling is driven by the accumulation of partial dislocations and stacking faults induced by high plastic deformation and nanocrystalline grain size [63,64,65,66,67]. In addition, the presence of diffraction peaks of YSZ is associated with the contamination that comes from the milling media.

### 3.2. Microstructural Characterization of Sintered Samples

**Figure 3:**
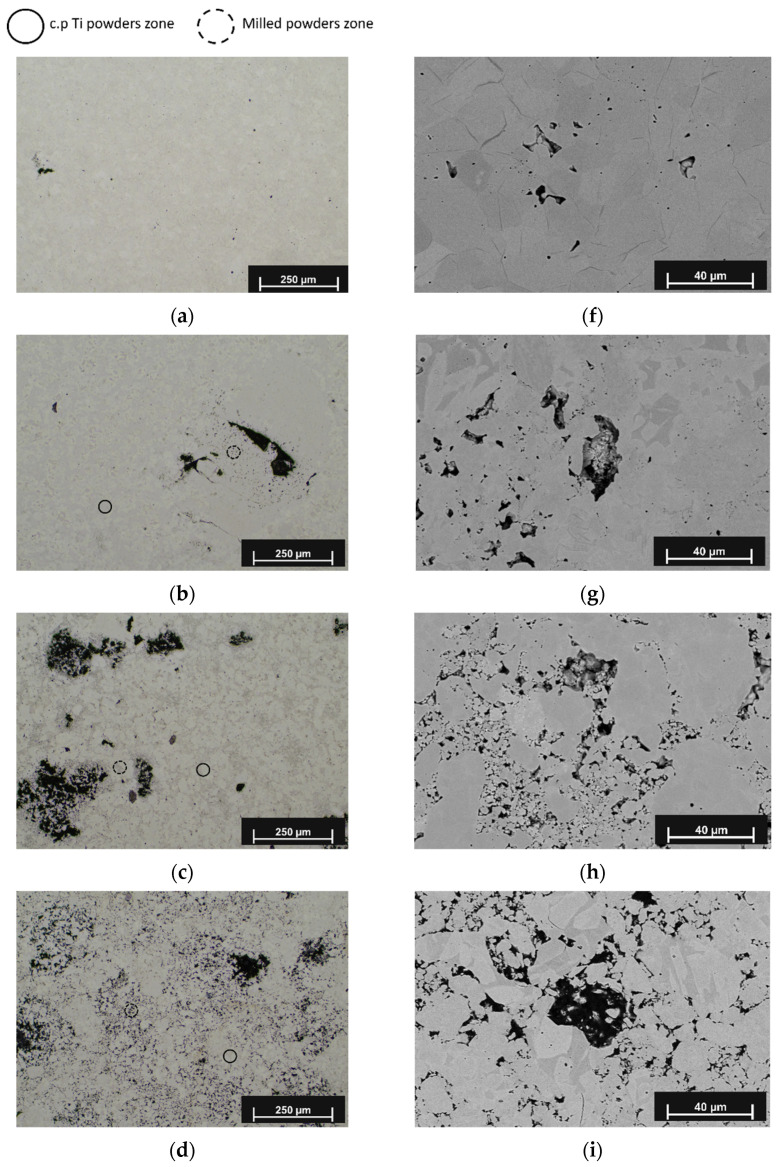

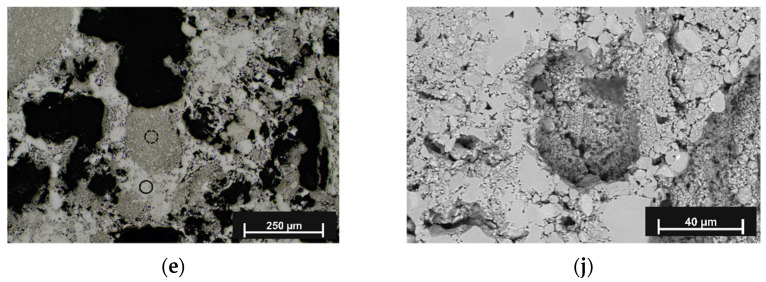
Optical micrographs of Ti samples: (**a**) c.p. Ti; (**b**) Ti_10–50_; (**c**) Ti_20–50_; (**d**) Ti_10–75_; (**e**) Ti_20–75_; BSE-SEM images of Ti foams: (**f**) c.p. Ti; (**g**) Ti_10–50_; (**h**) Ti_20–50_; (**i**) Ti_10–75_; (**j**) Ti_20–75_.

The results from the optical microscopy of the obtained samples are shown in Figure 3. Figure 3a represents an optical microscopy of the c.p. Ti specimen with 50 vol.%. In this sample, a titanium matrix with a very low porosity is observed, with isolated pores with sizes close to 20 microns. According to the results, the porosity is well below that obtained with the conventional powder metallurgy technique, where 50% spacer generates porosities between 40% and 45% [59]. However, as indicated in Table 2, the porosity obtained is less than 2%. This means that hot pressing has eliminated both the spacer and the porosity that it could have generated, so that it would not be named a “metal foam” with a final porosity of 45–60% pores. In addition, in Figure 3b–e, the micrographs of the Ti_10_ and Ti_20_ samples are shown, respectively. In general, a porous matrix with a bimodal microstructure, with higher porosity compared to the c.p Ti, is observed. The addition of spacer particles and milled powders promotes a porous structure that exhibits irregular pores with rounded borders. These pores reach a mean size of between approximately 40 and 250 μm, and they are surrounded by a fine-particle structure; pores with a lower size are homogenously and randomly distributed throughout the titanium matrix. It is observed that the porosity percentage goes up as the milling time and the percentage of milled powders increase. It can be stated that porosity depends equally on both factors. An increase in the pore size with the milling time is also noticed, where the maximum pore sizes were achieved for those samples that contain the Ti_20_ powder. In addition, the porosity in the sample was affected by the amount of milled powder used to produce the bimodal microstructure, where the small pores go up when the milled powder percentage increases. 

The differences in pore size could be a consequence of the particle size distribution of the used powders. In this sense, it is possible that smaller particles were used to redistribute its mass to larger particles [68]. If a small particle forms a neck with large particles and redistributes its mass with them, and these large particles were restricted in movement together (as is the case when surrounded by denser regions), then the smaller particle would break away (de-sinter) from one of the larger particles and be absorbed by the other. In consequence, pore growth takes place to reduce the surface to volume ratio of a powder compact when the compact is restricted from shrinking, as is the case for the bimodal microstructure, where pore growth occurs in regions where densification is locally restricted by the denser zones (unmilled powders), although these partially dense zones are globally subjected to densification particles [68]. Furthermore, it is known that agglomerates have a strong influence on densification because they prevent the effective transfer of heat and pressure to the particles during sintering [69,70,71,72]. Another de-sintering cause in the powder compact could be the presence of inclusions [73]. This behavior could be caused by the contamination of the ground powders during the milling process, caused by the release of ZrO_2_ from the milling medium. This lack of sintering is related to the fact that these contaminants are refractory materials [74]. Furthermore, the oxide content in the milled powders hinders the particle–boundary motion of coarse particles during sintering, hereby lowering the particle coalescence [75].

Backscattering Electron–SEM images (BSE-SEM) of sintered samples are shown in Figure 3f–j, where the microstructure of the titanium sample (Figure 3f) was compared with those compacts prepared from the mixed powders (Figure 3g–j). On one hand, in the sample of pure titanium with 50 vol.% NH_4_HCO_3_ (Figure 3f), a typical microstructure of c.p. Ti with equiaxial grains and some micropores in the matrix was observed. However, the spacer and the pores, which should have been generated from it, had disappeared due to the densification action of the HP process. On the other hand, the samples prepared from the mixed powders exhibited a bimodal particle microstructure consisting of coarse and fine grains, where the bimodal microstructure can be identified by the presence of clusters of microporosities, which are caused by the fine particles. The coarse particles that originate from the unmilled powders are randomly distributed and surrounded by porous regions with fine particle microstructure. The porosity of compact prepared from the mixed powders is higher than that observed in the c.p. Ti samples (Figure 3a,f). The porosity increases as the amount of milled powder and milling time increases. It should be noticed that only in Ti_20–75_ could the observed porosity values correspond to a porosity close to that added by the spacer. Relatively large pores in the order of 250 microns are observed in this sample (Figure 3e), but also small pores left by the spacer, as seen in Figure 3f. This figure presents and describes the surface of a pore caused by the spacer with a relatively small size, but it is also possible to distinguish the bimodal microstructure.

**Figure 4:**
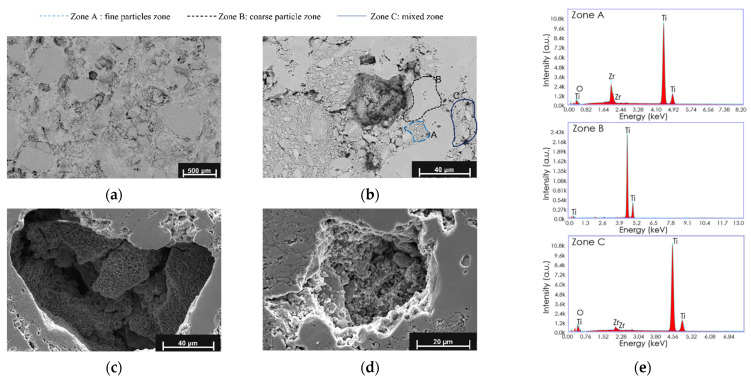
Microstructural aspects of synthesized Ti_20–75_ specimen: BSE-SEM images (**a**) general view of bimodal microstructure; (**b**) characteristic microstructure of sample with three zones: Zone A—fine particles; Zone B—coarse particle; Zone C—mixed zone. SE-SEM images (**c**) Macro-pore surface; (**d**) Micro-pore surface; (**e**) EDS analysis for the different zones present in bimodal structure: Zone A, Zone B, and Zone C.

Interesting aspects about the bimodal microstructure and porosity in Ti_20–75_ samples are presented in Figure 4. The bimodal microstructure is evenly distributed throughout the entire specimen (Figure 4a), which indicates that a good homogenization was achieved during the mixing process. Three zones were identified in the samples (Figure 4b) as follows: (i) Zone A: fine particles zone, where the milled powders are predominant, surrounding a coarse particle zone (Zone B); (ii) Zone B: coarse particle zone, promoted by unmilled powders; (iii) Zone C: an intermediate or mixed zone with both fine and coarse particles. The formation of the three zones can possibly be attributed to assembly mechanisms between the powder particles, forming clusters through agglomeration. Although different mechanisms lead to the agglomeration of particles in a sample, in this case, the agglomeration could occur during mechanical milling and even blending, where the particles collide and can stick together as a result of completely random movement within the confined space such as the grinding vessel or mixing vessel [76]. The agglomeration of the particles by size was observed in the particle size distributions for the titanium ground at 10 h and 20 h (Figure 1g,h). Another mechanism that can carry out the agglomeration of the particles during these milling or mixing events is known as gravitational agglomeration, which depends on the size of the particles and their speed, where the particles that settle slower are trapped by those that settle faster [76]. The latter may explain the mixed zone (Zone C, Figure 4). In addition, the matrix composition in the different zones present in the bimodal microstructure was evaluated by EDS analysis. The presence of ZrO_2_ is detected in Zones A and C, comprised by milled powders, which is the result of the contamination of the milling media.

Two types of porosity were distinguished, as is observed in the Secondary Electron–SEM (SE-SEM) images shown in Figure 4c,d: the porosities were promoted by the spacer particles with a pore size of around 100 µm, a rough surface, rounded borders, and were interconnected (Figure 4c), and the porosities inherent to the powder metallurgy process with a pore size of around 10–50 µm and a rough surface were surrounded by the Zones A and B (Figure 4d).

**Figure 5:**
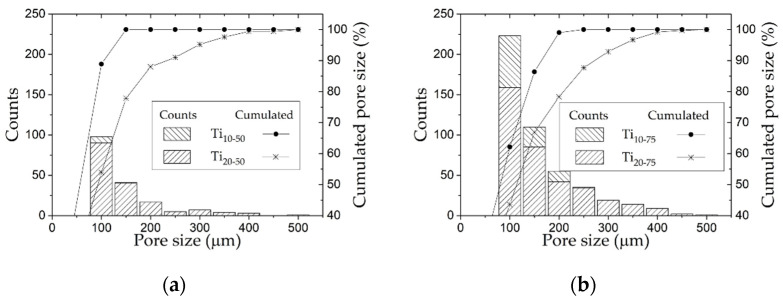
Pore size distribution: (**a**) Ti_10–50_, Ti_20–50_ (50% milled powder); (**b**) Ti_10–75_, Ti_20–75_ (75% milled powder).

The results of image analysis are summarized in Figure 5, where the stacked frequency distribution histograms of the pore size for each of the consolidated specimens are presented. Each bar of the histograms represents a quantity of pore counts with size in the comprised range. It is important to remember that all of the study samples in this graph have a 50% spacer added. The objective, then, is to evaluate the effect of the amount of ground powder and the grinding time on the pore size and on the porosity that could be obtained despite the use of the HP process. This figure shows the accumulated pore size corresponding to spacers with significant amounts of sizes between 100 and 150 microns, where it is possible to observe that specimens with higher amounts of fine powders (Ti_10–75_ and Ti_20–75_, Figure 5b) show higher frequencies of larger pores. The mentioned effect on porosity is observed in Figure 3d,e, where there are bigger pores in specimens using 75 wt.% of milled powders. A similar behavior has been documented by Dirras et al. [75], who produced Ni samples using different particle sizes and observed a “shielding effect” by introducing coarse particles onto a fine-particle matrix because the coalescence of coarse particles was hindered.

In addition, the pore size distribution shows that the samples Ti_10–50_ and Ti_10–75_ (10 h of milling time) exhibit porosities up to 200 µm, while the samples with the Ti_20_ powder have 80% of its total porosity in this range, and the remaining 20% is between 200 and 400 µm.

To complement this analysis of the porosity size, Table 2 presents the image analysis data, showing the porosity percentage, the mean equivalent diameter, and the shape factor values expressed in terms of mean value ± standard error. This value of porosity corresponds to that obtained only with the spacer. To do this, a criterion was applied where the pore size data was filtered, eliminating all the pores that were below 50 µm. Porosity percentage increases with the amount of milled powders, where the highest porosity was achieved for Ti_20–75_ (~36%). Although the porosity obtained is less than the percentage of the added spacer, 50% NH_4_HCO_3_, it is important to remember that the hot-pressing technique is used to obtain compacts with high densification. In this case, it is not only intended to introduce porosity in the consolidated titanium matrix, but it also seeks to improve the properties of the matrix and cycle times and reduce the temperature necessary for consolidation. The equivalent pore diameter was found in a range comprised between 84 ± 8 and 141 ± 4 μm. The ANOVA indicates that statistically significant differences exist in the pore size (*p*-value < 0.05), which confirms the suggested difference observed in the optical micrographs. There are influences of the milling time as well as of the amount of milled powder on the equivalent pore diameter in the specimens. By means of a Tukey’s test, it was determined that the greatest significant difference of the equivalent diameter is about 60 μm. The pores shape factor was found in a range comprised between 0.70 and 0.80. In this case, the ANOVA indicates that there are no statistically significant differences (*p*-value > 0.05). This behavior is attributed to the deformation strengthening mechanism, where the milled powders have lost their ductile behavior; thus, the deformation is carried out mainly by the coarse particles. Another cause is the reduced size of the particles that have a greater surface energy, which results in a faster sintering that hinders the rearrangement of particles along the matrix.

### 3.3. Mechanical Characterization of Sintered Samples

**Figure 6:**
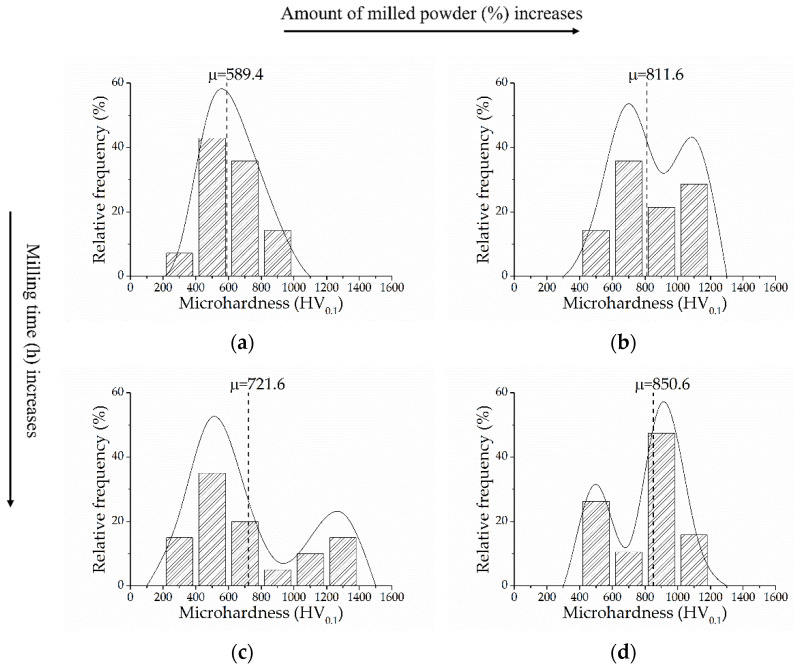
Vickers microhardness distribution for samples: (**a**) Ti_10–50_; (**b**) Ti_10–75_; (**c**) Ti_20–50_; (**d**) Ti_20–75_.

Vickers microhardness distribution found in the consolidated samples with bimodal microstructure is shown in Figure 6. The mean values of microhardness measurements ranged between 589.4 ± 43.3 HV and 850.6 ± 62.2 HV, where the highest is found for Ti_20–75_. These values exceed the c.p. Ti microhardness mean value (301 HV), being even higher than the reported values for the c.p. titanium components with a nanocrystalline structure or ultrafine-grained components consolidated by SPS [77], high-pressure torsion [78], or multi-pass ECAP [79], which are comprised in a range of 230 to 250 HV. They are closer to the ones reported for titanium components with a bimodal microstructure consolidated by SPS (660 to 853 HV) [45]. As it is observed, the microhardness distribution varies in function of the processing parameters: (i) when the amount of milled powder increases, the frequency of higher microhardness values increases, while (ii) the longer the milling time, the higher the mean value of microhardness. This increase in microhardness with the milling time and quantity of ground powder is mainly attributed to the accumulation of deformation energy [80], but also it could be influenced by the contamination with the milling medium, which in turn depends on the time, intensity, atmosphere of milling, and the difference in the strength/hardness of the powders and milling medium [81]. Table 3 summarizes the statistical analysis of each microhardness distribution. From the ANOVA, it was determined that both parameters have a significant effect (*p*-value < 0.05) on the microhardness value. However, the amount of ground powder is more significant in increasing the microhardness, as shown in Table 3.

In addition, the mechanical properties estimated from the porosity and shape factor obtained from the image analysis are summarized in Table 4. These values are expressed in terms of mean value ± standard error. As it is observed, the elastic modulus decreases as the milling time and/or content of the milled powder increases, following the trend of increasing porosity. Yield strength has similar behavior due to the fact that it depends on the density value, and this is directly related to the porosity level. Nonetheless, the yield strength was estimated from a model which does not consider the strengthening effect due to deformation during the milling stage of the powders, or the presence of a bimodal structure, hence it is necessary to develop models in order to predict this behavior, which can be contrasted with experimental data. 

Previous studies have determined that the optimal porosity for an implant to efficiently stimulate bone ingrowth is in the range of 20–50% [82] with a pore size of 100–400 µm [83]. Furthermore, taking into account that the elastic modulus of the cortical bone is in a range from 20 to 25 GPa and that its ultimate tensile strength is about 195 MPa [3], it is ascertained that the Ti_20–75_ specimen shows the characteristics suitable for bone replacement applications. The fatigue behavior of the specimens presented in this work is planned for future experiments, since metal fatigue [84] is one of the main causes of implants’ mechanical failure.

## 4. Conclusions

In this study, the porous titanium samples with a bimodal microstructure were successfully synthesized by hot pressing with NH_4_HCO_3_ as a space-holder. The effects of the milling time for obtaining fine powder as well as its amount over the microhardness and porosity were investigated. The conclusions are shown as follows:

- It has been determined that the processing route via the space-holder technique and hot pressing consolidation is effective in producing titanium samples with a porosity of 36% with a bimodal microstructure, whose microhardness has similar values to those obtained in nanocrystalline or ultrafine-grained microstructures synthesized by SPS. The microhardness value depends on the amount of fine powder that constitutes the matrix, and it is the result of deformation strengthening mechanisms and a small grain size;
- The obtained porosity in the titanium samples processed by this route depends on the milling time as well as the amount of fine powder due to the changes in particles size and distribution, deformation/rearrange capability of the powders, and the presence of agglomerates and contamination, all parameters which affect the compaction and sintering processes. However, when the milled powders for 20 h were used, larger pores were reached, whose sizes reached up to 400 µm. The Ti_20–75_ sample presents appropriated mechanical properties for cortical bone replacement applications.

## Figures and Tables

**Table 1:** Powder parameters for blend processing.

Milling Time (h)	Nomenclature	Portion of Milled Powder (wt.%)	Nomenclature
10	Ti_10_	50	Ti_10–50_
		75	Ti_10–75_
20	Ti_20_	50	Ti_20–50_
		75	Ti_20–75_

**Table 2:** Morphological parameters of pores.

Sample Name	*P*(IA) (%)	*D_eq_* (µm)	*F_f_*
Ti_10–50_	1.5 ± 0.4	84 ± 8	0.70 ± 0.16
Ti_20–50_	12.5 ± 3.8	121 ± 6	0.75 ± 0.11
Ti_10–75_	13.2 ± 0.4	100 ± 4	0.75 ± 0.07
Ti_20–75_	35.5 ± 1.5	141 ± 4	0.80 ± 0.09

**Table 3:** Summary of statistical analysis of microhardness values (HV).

Sample	Milling Time (h)	Milled Powder (%)	Porosity (%)	Max. HV	Min. HV	Mean HV	Std. Error HV
c.p. Ti	0	0	-	430.8	232.1	312.9	11.2
Ti_10–50_	10	50	1.5 ± 0.4	890.3	374.5	589.4	43.3
Ti_20–50_	20	50	12.5 ± 3.8	1348.6	227.3	721.6	75.7
Ti_10–75_	10	75	13.2 ± 0.4	1109.4	516.7	811.6	52.5
Ti_20–75_	20	75	35.5 ± 1.5	1674.5	456.9	850.6	62.2

**Table 4:** Estimated mechanical properties from bulk properties by means of Nielsen’s method [56] (*E_p_*) and Jha’s correlation [58] (*σ_y,f_*).

Sample	*E_p_* (GPa)	*σ_y,f_* (MPa)
Ti_10–50_	106.0 ± 0.2	312.8
Ti_20–50_	80.9 ± 8.4	314.5
Ti_10–75_	79.4 ± 1.5	317.8
Ti_20–75_	41.8 ± 4.1	234.1

## Data Availability

The data presented in this study are available on request from the corresponding author.
